# In Vitro Studies on Therapeutic Effects of Cannabidiol in Neural Cells: Neurons, Glia, and Neural Stem Cells

**DOI:** 10.3390/molecules26196077

**Published:** 2021-10-08

**Authors:** Jungnam Kim, Hyunwoo Choi, Eunhye K. Kang, Gil Yong Ji, Youjeong Kim, Insung S. Choi

**Affiliations:** 1 Department of Chemistry, KAIST, Daejeon 34141, Korea; wjdska13@kaist.ac.kr (J.K.); hwchoi0806@kaist.ac.kr (H.C.); ehkang@kaist.ac.kr (E.K.K.); 2 Cannabis Medical, Inc., Sandong-ro 433-31, Eumbong-myeon, Asan-si 31418, Korea; gyji@osungam.com (G.Y.J.); cm@osungam.com (Y.K.)

**Keywords:** cannabidiol, neural cells, neurological disorder, neuroprotection, therapeutic effect

## Abstract

(‒)-Cannabidiol (CBD) is one of the major phytocannabinoids extracted from the *Cannabis* genus. Its non-psychoactiveness and therapeutic potential, partly along with some anecdotal—if not scientific or clinical—evidence on the prevention and treatment of neurological diseases, have led researchers to investigate the biochemical actions of CBD on neural cells. This review summarizes the previously reported mechanistic studies of the CBD actions on primary neural cells at the in vitro cell-culture level. The neural cells are classified into neurons, microglia, astrocytes, oligodendrocytes, and neural stem cells, and the CBD effects on each cell type are described. After brief introduction on CBD and in vitro studies of CBD actions on neural cells, the neuroprotective capability of CBD on primary neurons with the suggested operating actions is discussed, followed by the reported CBD actions on glia and the CBD-induced regeneration from neural stem cells. A summary section gives a general overview of the biochemical actions of CBD on neural cells, with a future perspective. This review will provide a basic and fundamental, but crucial, insight on the mechanistic understanding of CBD actions on neural cells in the brain, at the molecular level, and the therapeutic potential of CBD in the prevention and treatment of neurological diseases, although to date, there seem to have been relatively limited research activities and reports on the cell culture-level, in vitro studies of CBD effects on primary neural cells.

## 1. Introduction

(‒)-Cannabidiol (2-[(1*R*,6*R*)-3-methly-6-prop-1-en-2-ylcyclohex-2-en-1-yl]-5-pentylbenzene-1,3-diol, CBD) is a major non-psychoactive phytocannabinoid derived from the *Cannabis* genus. CBD has emerged as a ray of hope for neurological disorders in pharmacological and medicinal fields, because of its deep and wide potential of therapeutic effects including neuroprotective, anti-inflammatory, anti-epileptic, anxiolytic, anti-depressant, and even anti-addictive effects [1,2,3,4,5]. CBD also has received great attention as a potential therapeutic candidate for multifactorial neurological diseases, the exact cause of which is hard to determine, because in addition to the non-psychoactive characteristics, unlike other potentially therapeutic but psychoactive cannabinoids (e.g., tetrahydrocannabinol (THC)), CBD has so far been reported to have multiple molecular targets, including ion channels (e.g., TRPV1, TRPV2, and L-type VGCC), receptors (e.g., CB1, CB2, GPR55, PPARγ, 5-HT1A, and A2A receptors), transporters (e.g., anandamide membrane transporter), and enzymes (e.g., FAAH and MAGL) [6,7].

CBD has been shown to exhibit better safety profiles than other cannabinoids (e.g., THC), as exemplified by the tolerance of high doses up to 1500 mg/day in humans, and no changes in heart rate, blood pressure, and body temperature after CBD intake in humans and rodents [2,6]. Furthermore, its side effects in the adjunctive treatment for schizophrenia are not noticeably different from the placebo [8,9]. On the other hand, some pre-clinical and clinical trials with CBD for epilepsy and non-epilepsy have showed serious or mild adverse effects in humans, including abnormal liver functions, pneumonia, decreased appetite, diarrhea, sedation, fatigue, vomiting, and somnolence, as well as developmental toxicity, CNS inhibition (e.g., depression, sedation, and prostration), organ weight elevation (e.g., liver and kidney), hepatic abnormality, reproductive dysfunction (e.g., testicular weight decrease), at high doses of CBD, in animals [10,11,12,13]. Therefore, comprehensive and scientific research to disclose the exact actions of CBD in vivo, such as drug–receptor and drug–drug interactions, should be executed to ensure safety for the therapeutic use of CBD.

Despite the intensive studies on the pharmacological effects of CBD, precise biochemical mechanisms on the CBD treatment for brain disorders still remain to be seen. A majority of the research have been based on the preclinical studies with animals and the small-scale clinical studies [9,14], and the mechanistic complexity of CBD actions has made the elucidation of CBD’s biological profiles challenging and daunting; CBD is reported to work in different biological pathways depending on the physical or pathological conditions and even the identity of pathogens [7,15]. For example, CBD inhibits the LPS-induced microglial activation by activating PPARγ in the culture of primary microglia derived from mice [16], but it attenuates the microglial activation by the A2A receptor in the viral infection-induced mice model [17]. It is difficult to elucidate the mechanistic action of CBD on each and every cell type in the CNS, because CNS is an extremely complex circuitry comprised of various cell types that communicate with each other, including neurons and glia. Therefore, it is necessary to delicately design the in vitro experimental protocols, which would allow for the tight regulation of the control variables proposed to affect the CBD actions.

Two types of neurocellular models have mainly been used for in vitro culture experiments: primary neural cells and continuous neural cell lines. The continuous neural cell line, an immortalized cell or a tumor-derived cell expressing a phenotype for the cell of interest, has widely been used in neuroscience studies because of certain benefits, including genetic homogeneity and easy-handling procedure [18,19]. However, the continuous neural cell line could not faithfully and entirely substitute the in vivo neural cells in tissue, due to its inconsonant characteristics; for example, it does not form definite axons, dendrites, and synapses, which play important roles in the functions of neural cells in vivo [18,19]. In addition, it has rather different biomolecular profiles from neural cells in vivo for neurotransmitters, receptors, and proteins [18]. For example, HT22 and PC12 cells, which are both extensively used neuronal cell lines, have been observed to express a negligible amount of the CB1, which is abundant in neurons in vivo [20,21]. On the other hand, primary neural cells obtained directly from tissues (e.g., in the brain or spinal cord) outstandingly represent natural neural cells in the tissue of their origin with comparable morphological, physiological, and functional features [18,22]. However, some experimental challenges in the handling of primary neural cells, such as cell-type heterogeneity, practical insufficiency of materials (e.g., proteins and mRNAs) for biochemical analysis, finite generation or division impossibility in the case of mature neurons, and source dependency of genetic profiles [18,19], have led to limited but highly informative reports on the pharmacological potential of CBD on primary neural cells (e.g., neurons, microglia, astrocytes, oligodendrocytes, and neural stem cells). 

**Figure 1:**
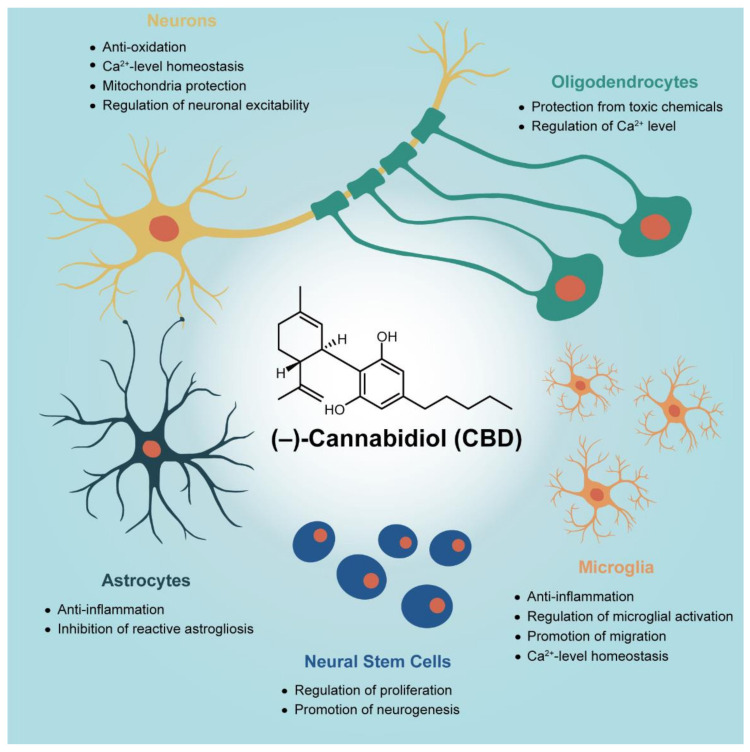
Reported CBD actions on neural cells.

This review provides an overview of the therapeutic effects of CBD, with a particular focus on primary neural cells in the in vitro culture experimentation. The sections are divided, based on cell types, into three parts—neurons, glia, and neural stem cells (Figure 1). The neuron section (Section 2) primarily discusses the neuroprotective effects of CBD against various pathological situations, which are frequently observed in neurological disorders, with the suggested biochemical actions of CBD (e.g., attenuation of reactive oxygen species (ROS) over-production, intracellular calcium (Ca^2+^) homeostasis, protection against mitochondrial dysfunction, and recovery of abnormal neuronal activities). The glia section (Section 3), with subsections for microglia, astrocytes, and oligodendrocytes, summarizes the reported therapeutic potential of CBD targeted at primary glial cells, including the inhibitory action of gliosis and inflammation in microglia or astrocytes, and the protective action on oligodendrocytes against pathological environments. The neural stem cell section (Section 4) deals with the regulatory actions of CBD in neural stem cells (NSCs), which was proposed based on in vivo and in vitro studies, such as the promotion of survival, proliferation, and neural differentiation/maturation of NSCs in physiological conditions, and the protection of NSCs against pathological environments. If the therapeutic effect of CBD and its mechanism are identified accurately for each cell type in CNS, we can expect a synergistic effect and guaranteed safety while minimizing the unnecessary side effect by using CBD appropriately to the cell’s condition in CNS. A future perspective on the fundamental research of CBD effects on neural cells and its contribution to the medical *Cannabis* sector is also provided.

## 2. Neuroprotection of CBD on Primary Neurons

**Figure 2:**
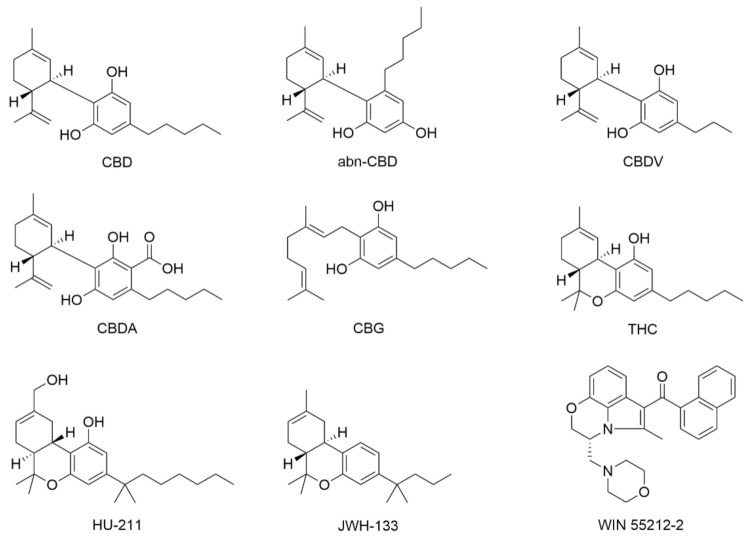
Chemical structures of cannabinoids and their synthetic analogs discussed in this review.

**Figure 3:**
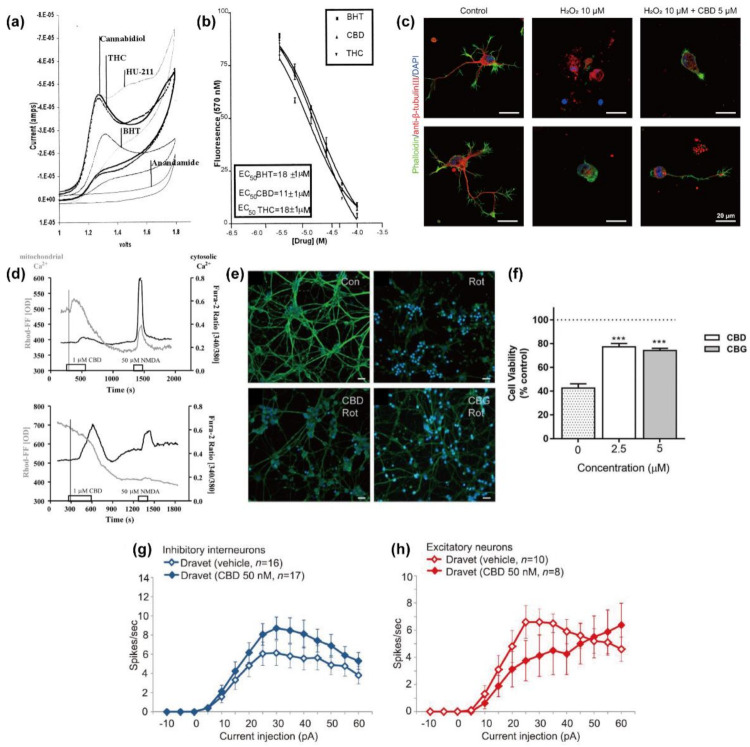
CBD actions on neurons. (**a**) A comparison of the oxidation potentials of cannabinoids and the antioxidant BHT. (**b**) Effects of CBD and BHT on prevention of *tert*-butyl hydroperoxide-induced oxidation of dihydrorhodamine. (**a**,**b**) Reproduced with permission from [23]. Copyright 1998, National Academy of Sciences, U.S.A. (**c**) High-magnification CLSM images of hippocampal neurons after 24 h treatment of (**middle**) H_2_O_2_ (10 µM), (**right**) H_2_O_2_ (10 µM), and CBD (5 µM). The cells were stained green (F-actin), red (β-tubulin III), and blue (nuclei). Reproduced from [24]. (**d**) Effects of CBD (1 µM) on mitochondrial and cytosolic Ca^2+^ responses of hippocampal neuron cultures, loaded with Rhod-FF, AM, and fura-2 AM. Reproduced with permission from [25]. Copyright 2009, Society for Neuroscience. (**e**) Immunocytochemistry images of the CGNs pre-treated with CBD (2.5 µM; 1 h) or CBG (5 µM; 1 h) and exposed to rotenone. The cells were stained with Hoechst 33258 dye (blue; nuclei) and the cytoskeleton marker anti-α-tubulin (green). Scale bar: 20 µm. (**f**) Neuroprotective effects of CBD (1 h pre-treatment) against rotenone. The statistical analysis was performed using one-way ANOVA; *** *p* < 0.001 for CBD (2.5 µM) and CBG (5 µM) versus rotenone exposure. (**e**,**f**) Reproduced with permission from [26]. Copyright 2020, Springer Science Business Media, LLC, part of Springer Nature. (**g**,**h**) Comparison of the action potential firing between the control group and the 50 nM CBD-treated group for (**g**) inhibitory neurons and (**h**) excitatory neurons from Dravet syndrome patient-derived iPSCs. Reproduced from [27], under the terms of the CC-BY Creative Commons Attribution 4.0 International license (https://creativecommons.org/licenses/by/4.0/, accessed on: 15 August 2021).

CBD (Figure 2), as a therapeutic candidate, has been suggested to possess neuroprotective properties in various cellular models of neurological disorders, including Alzheimer’s disease (AD), Parkinson’s disease (PD), amyotrophic lateral sclerosis (ALS), and multiple sclerosis (MS). A multitude of different molecules or receptors have been identified as biochemical targets of CBD. However, CBD has been reported to show somewhat conflicting behaviors depending upon toxic agents and pathological conditions, which makes it difficult to lucidly elucidate the biochemical mechanisms on the neuroprotective properties of CBD. This section provides an overview of the neuroprotective mechanisms of CBD with discussion on the previously reported biochemical roles of CBD in primary neurons, such as anti-oxidation, intracellular Ca^2+^ homeostasis, modulation of mitochondrial stress, and restoration of neural network activity (Figure 3 and Table 1).

### 2.1. Anti-Oxidation

Oxidative stress, a pathological condition commonly observed in various neurodegenerative diseases, is generally caused by the concentration imbalance between pro-oxidants and anti-oxidants [43]. Excess ROS are reported to induce apoptotic cascades and cause a massive loss of neurons via the impairment of intracellular Ca^2+^ homeostasis or the oxidatively modified lipid, which activates the MARK1-related signaling pathway. Anti-oxidants have widely been known to have neuroprotective ability against oxidative stress under a variety of neurotoxic conditions by inactivating highly reactive radicals.

The phenolic hydroxyl (-OH) group, a common functional group in the phytocannabinoids, serves as a good hydrogen donor [44]. In addition, the radicals that are generated from the phenolic group are chemically more stable than the radicals from reactive oxygen because of electron delocalization with the π-electrons of the benzene ring. Therefore, the reactions of the hydrogen-donating phenolic OH group and ROS could terminate the uncontrolled continuous generation of new radicals in the chain reactions. In addition to the direct radical-scavenging ability, the anti-oxidizing action of CBD in the cellular system would involve other mechanisms, such as interactions with specific receptor-mediated pathways or receptor-independent intracellular signaling pathways [7]. 

To investigate whether the anti-oxidizing property of CBD was of the chemical structure- and/or specific receptor (e.g., CB1)-mediated actions, Marsicano et al. performed the cell-free biochemical assays on the anti-oxidizing capability of various cannabinoids, including phyto-, endo-, and synthetic cannabinoids [21]. The cannabinoids were categorized based on the involvement of phenolic group and the potent ability for interactions with CB1. They found that the only compounds that contained the phenolic group in their structures, such as CBD, exhibited the anti-oxidizing capability, regardless of their acting as a ligand for CB1. In addition, CBD (10 µM) could almost completely inhibit the ascorbate-induced oxidation of rat brain lipids [45], and it could also reduce the copper sulfate-induced LDL oxidation in the human blood plasma by about 90% [46]. 

Hampson et al. reported that CBD had the oxidation potential comparable to butylhydroxytoluene (BHT), a well-known anti-oxidant, based on cyclic voltammetry analysis, measuring the ability of a compound to accept or donate electrons, and Fenton reaction-based analysis, measuring the ability of a compound to inhibit the oxidation of dihydrorhodamine under iron-catalyzed, ROS-generating conditions (Figure 3a,b) [23]. The anti-oxidizing ability of CBD also had a similar level to another phytocannabinoid found in *Cannabis*, THC, and a synthetic cannabinoid, HU-211, which share core chemical structures with CBD (Figure 2). The report suggested that a certain chemical moiety in CBD, such as the phenolic group, might play a critical role in its anti-oxidizing action. They showed that CBD (1–31.6 μM) protected primary cortical neurons from ROS-induced toxicity, generated by *tert*-butyl hydroperoxide, with EC_50_ of 6.2 µM. CBD (10 µM) also diminished the glutamate neurotoxicity to the cortical neurons by 60%, which is more effective than ascorbate (vitamin C) and α-tocopherol, which are common natural ROS scavengers in the cellular system.

Echeverry et al. have recently reported the neuroprotective effect of CBD against hydrogen peroxide (H_2_O_2_), a pivotal mediator of cellular oxidative stress, for primary cerebellar granule neurons (CGNs) [26]. Pre-incubation of CGNs with CBD (2.5 µM; for 24 h) increased the cell viability to 54% from 41% for the H_2_O_2_-treated CGNs (protective ratio (PR): 1.33). CBD was also found to be more effective in the neuroprotection than another non-psychoactive phytocannabinoid, cannabigerol (CBG) (Figure 2). Of interest, the 1 h pre-incubation with CBD was more effective (PR: 1.75) than the 24 h pre-incubation, suggesting that the direct anti-oxidant reaction, such as free radical capture, was predominant in the neuroprotective mechanism of CBD against H_2_O_2_. On the other hand, the 1 h pre-incubation of neuroblastoma SH-SY5Y cells with CBD did not lead to noticeable protection of the cells against H_2_O_2_, while the direct co-application of CBD and H_2_O_2_ to the cells showed the slightly protective effect of 16.5% [25]. 

Kim et al. investigated the concentration-dependent neuroprotective ability of CBD against H_2_O_2_ for primary hippocampal neurons [24]. The 24 h treatment of hippocampal neurons, from rat embryos, with H_2_O_2_ (10 µM) decreased the cell viability to 24%, but CBD (5 µM) protected the neurons from H_2_O_2_, and the viability greatly increased to 57% (PR: 2.40). In this study, the neurons were treated simultaneously with CBD and H_2_O_2_ for neuroprotection; the pre-incubation with CBD (5 µM, 1 h) was not effective, which is similar to the result for the SH-SY5Y cells described above [25]. They also observed that the oxidative stress-related morphological changes of the neurons, such as shrunken nuclei and neurite debris, were inhibited in part by CBD, suggesting that CBD might serve as a neuroprotective anti-oxidant against the apoptotic progress induced by oxidative stress (Figure 3c).

### 2.2. Homeostasis of Intracellular Ca^2+^ Level

Intracellular Ca^2+^ signaling in the neurons plays an important biochemical role in the regulation of neuronal excitability by the action of Ca^2+^ as a second messenger for signal delivery to the postsynaptic cells in response to the synaptic inputs that include the membrane depolarization caused by the changes in the membrane potential [47,48]. The intracellular calcium level ([Ca^2+^]_i_) has been reported to be controlled mainly by the ion flux, via plasma membrane receptors, voltage-gated ion channels, and the intracellular storage in the organelles, such as mitochondria and ER [49]. The intracellular Ca^2+^ signaling also induces the ATP consumption and the mitochondrial generation of ROS [50]. Therefore, the dysregulation of intracellular Ca^2+^ homeostasis might be a trigger of neurodegenerative conditions; it has widely been observed as one of the characteristics in the neurological disorders. 

#### 2.2.1. Mitochondria

Platt et al. studied the regulatory roles of CBD in the intracellular Ca^2+^ signaling by quantifying the [Ca^2+^]_i_ changes upon the CBD treatment of mature hippocampal neurons under various conditions [25,28,30,31]. CBD (1 µM) increased the [Ca^2+^]_i_ of the neurons in the physiological buffer solution (HEPES-based solution that contained 5.4 mM of KCl, 1.8 mM of CaCl_2_, 1 mM of MgCl_2_, 130 mM of NaCl, and 25 mM of D-glucose) by about 45% compared with the basal [Ca^2+^]_i_, and any [Ca^2+^]_i_ increase-induced neurotoxicity was not observed for 48 h [28]. The CBD-induced increase in [Ca^2+^]_i_ tended to persist even after cell washing, and only 19% of the neurons fully recovered [Ca^2+^]_i_ after two consecutive CBD treatments with an interval of 15 min. In addition, the [Ca^2+^]_i_ increase in the CBD-treated neurons was not blocked completely by either cadmium, a non-specific VGCC blocker, or nifedipine, a selective blocker of L-type VGCC; in both cases, the [Ca^2+^]_i_ increase was measured to be 18%. These results suggested that the CBD-induced [Ca^2+^]_i_ increase in hippocampal neurons might involve the Ca^2+^ release from the intracellular stores (e.g., mitochondria and ER) as well as the calcium gradient and L-type VGCC. Another report showed that CBD (10 µM) led to the immediate [Ca^2+^]_i_ increase even in the Ca^2+^-free saline, supporting the potential participation of the intracellular stores in the [Ca^2+^]_i_ regulation of CBD [31]. The degree of CBD-induced [Ca^2+^]_i_ increase was reduced in the highly excitatory buffer that contained Ca^2+^ and potassium (K^+^) ions 3–3.5 times more than the standard buffer, implying that the extracellular space might not be the major source in [Ca^2+^]_i_ regulation by CBD.

Ryan et al. reported that the CBD treatment of hippocampal neurons upregulated the [Ca^2+^]_i_ with the transient or delayed downregulation of mitochondrial Ca^2+^ concentrations, based on the co-imaging data made with a cytosolic Ca^2+^ indicator, fura-2 AM, and a mitochondrion-specific Ca^2+^ sensor, Rhod-FF, AM (Figure 3d) [25]. In addition, carbonyl cyanide-4-(trifluoromethoxy)phenylhydrazone (FCCP), an uncoupler of ATP synthesis causing the depletion of mitochondrial Ca^2+^, completely restrained the CBD-induced [Ca^2+^]_i_ elevation, which suggested that mitochondria might act as a dominant source for the CBD-based regulation of Ca^2+^ homeostasis. The CBD-induced [Ca^2+^]_i_ increase was also prohibited by CGP-37157, which is an inhibitor of the mitochondrial Na^+^/Ca^2+^ exchanger, but not by cyclosporine A, which is an mPTP inhibitor. In addition, the pre-incubation of the neurons with dantrolene and 2-APB, antagonists of ER-related ryanodine and IP_3_ receptors each, did not influence the [Ca^2+^]_i_ elevation. Taken together, the results indicated that the mitochondrial Na^+^/Ca^2+^ exchanger, not the ER receptor, might play a critical role in the [Ca^2+^]_i_-regulatory action of CBD in hippocampal neurons. They also showed that CBD supported the maintenance of the [Ca^2+^]_i_ homeostasis by attenuating the [Ca^2+^]_i_ increase, in the over-excitatory condition made by the double concentration of K^+^, by about 26%. Moreover, CBD was observed to act as a neuroprotective agent by pacifying the [Ca^2+^]_i_ rise and oscillation under the epileptic conditions that were induced by 4-AP, which is a blocker of the K^+^ channel. CBD also could protect the neurons from the mitochondrial dysfunction caused by oligomycin.

#### 2.2.2. Endoplasmic Reticulum (ER)

In addition to the reports on the mitochondrion-mediated [Ca^2+^]_i_ regulation of CBD, other studies indicated that ER, generally known as a central player in [Ca^2+^]_i_ homeostasis, also might be involved in the action [51]. For example, the [Ca^2+^]_i_-elevating capability of CBD (1 µM) was blocked fully on average, when hippocampal neurons were pre-treated with thapsigargin (Tg) (2 µM), an irreversible blocker of ER Ca^2+^-ATPase, which causes the depletion of Ca^2+^ in ER and prevents any subsequent occurrence of ER-dependent responses [28]. Another study showed that the pre-treatment of cortical neurons with CBD (10 µM) reduced the [Ca^2+^]_i_ increase that was induced by Tg (200 nM) [31]. The CBD effect was concentration-dependent: the 3-min treatment of the cells with CBD (10 µM) reduced the Tg-evoked Ca^2+^ release from ER by about 40% on average, but CBD at lower concentration (1 µM) did not. In addition, they also reported that CBD depressed SOCE, which was developed by addition of Ca^2+^ (2 mM) to the Tg-treated system. Taken together, these results supported that the effect of CBD on [Ca^2+^]_i_ might be associated with the actions of ER as a source of Ca^2+^. Relatedly, NAGly, an endogenous cannabinoid, which also depressed SOCE, induced the increase in cytosolic zinc (Zn^2+^) level, implying the potential regulation of CBD on the Zn^2+^ signaling, which is associated with learning and memory defects [52].

#### 2.2.3. Endogenous Cannabinoid Systems

It was reported that the [Ca^2+^]_i_-regulatory action of CBD was also involved in the endogenous cannabinoid system. CBD was known to upregulate the endogenous *N*-arachidonoylethanolamine (AEA) level by preventing its re-uptake and hydrolysis [53], and the endocannabinoids, such as AEA and 2-arachidonoylglycerol (2-AG), increased [Ca^2+^]_i_ in several cellular models [54,55,56]. Ryan et al. exogenously added AEA or 2-AG to the hippocampal neuron culture or made endocannabinoids endogenously upregulated by the addition of glutamate [31]. They observed that CBD (1 µM) alone increased [Ca^2+^]_i_ by 45%, but the increase dropped to 22% or 12%, respectively, upon the co-addition of AEA or 2-AG with CBD. In addition, the pre-treatment of the neurons with glutamate decreased the CBD-induced [Ca^2+^]_i_ elevation to 26% (from 45%). The results suggested that the endocannabinoid-dependent effect of CBD on [Ca^2+^]_i_ might involve the direct, inhibitory action of the endocannabinoids and/or the activation of specific receptors, but the experiment showed that the increased level of the endocannabinoids, which was made by the addition of enzyme inhibitors of AEA-hydrolyzing FAAH and 2-AG-hydrolyzing MAGL, did not alter the [Ca^2+^]_i_ increase induced by CBD. 

Drysdale et al. reported that the antagonists of CB1 (i.e., AM281) and TRPV1 (i.e., CPZ) enhanced the CBD-induced [Ca^2+^]_i_ increase in hippocampal neurons, which implied that the CBD action on [Ca^2+^]_i_ was regulated negatively by CB1 and TRPV1 [28]. In addition, *Pertussis* toxin (PTX), an uncoupler of the G_i/o_-linked GPCR, did not reduce the CBD-induced [Ca^2+^]_i_ increase, while the CBD-induced [Ca^2+^]_i_ response was greatly increased to 312% from 45% by a PLC inhibitor (U73122) [31]. The results suggested that the G_q/11_/PLC signaling might be related negatively with the [Ca^2+^]_i_-modulatory action of CBD. 

On the other hand, Ryan et al. reported that CBD could synergistically assist THC to maximize the effect on [Ca^2+^]_i_ increase in hippocampal neurons [29]. While the [Ca^2+^]_i_ enhancement was 45% or 58% by CBD or THC only, respectively, the 1:1 mixture of CBD and THC increased [Ca^2+^]_i_ by 189% (for the mixture of purified compounds) and 314% (for the mixture of extracted compounds) with highly increased proportion of the responsive cells. CBD and THC seemed to work on the [Ca^2+^]_i_ regulation via different mechanisms, but the synergistic action could support the reports that *Cannabis* extracts had additional benefits in several clinical studies [57,58].

### 2.3. Protection against Mitochondrial Dysfunction

Mitochondria, one of the most important organelles in neurons, have vital roles, including ATP production, response to oxidative stress, and [Ca^2+^]_i_ homeostasis. Correspondingly, mitochondrial dysfunction has been known to be related with various aging-induced neurodegenerative diseases, such as AD, PD, and ALS [59]. In studies with neurodegenerative cellular models, the inhibitors of mitochondrial complex I, such as 1-methyl-4-phenylpyridinium (MPP) and rotenone, have often been used to chemically cause mitochondrial dysfunction [33]. 

Moldzio et al. reported that CBD could rescue the dopaminergic neurons from MPP-induced mitochondrial dysfunction [32]. While the number of dopaminergic neurons reduced to 45% by the treatment of MPP, compared with the non-treated neurons, the neuronal survival increased to 54% under the concomitant treatment with CBD (10 µM). In addition, MPP-induced damages on neuronal morphology, such as shrunken cell bodies and degenerated neurites, could be attenuated by CBD, with no effects on neurite number and length. 

CBD also protected primary CGNs against mitochondrial dysfunction [26]. In the study, the mitochondrial dysfunction was induced by rotenone, an inhibitor of mitochondrial electron transport chain, which causes the imbalance of energy metabolism by decreasing the ATP level. Pre-treatment of the cells with CBD (2.5 µM) reduced the rotenone-induced neurotoxicity, with a 1.83-fold increase in viability (Figure 3e,f). They also reported no differences in viability under rotenone-induced toxicity for 1 h and 24 h of CBD pre-treatment, which is different from the results on the CBD effects on H_2_O_2_, showing the higher neuroprotection with 1 h CBD pre-treatment than 24 h pre-treatment [26]. These results suggested that the neuroprotective action of CBD might be operational through different mechanisms depending upon toxin-induced cellular damages. It was additionally found that CB1/2 receptor and 5-HT1A receptor were not involved in the neuroprotection of CBD against rotenone, indicating that CBD might prevent neuronal death from the mitochondrial impairment through anti-oxidizing and other receptor-independent pathways, such as maintenance of [Ca^2+^]_i_ homeostasis. However, cannabidiolic acid (CBDA) (Figure 2), an acid form of CBD that shares a common structure and the antioxidant capacity with CBD, exhibited less, if any, effects than CBD against the neurotoxicity of rotenone, suggesting that CBD-specific interactions with intracellular signaling might potentially be involved in the neuroprotective process of CBD in this case. 

Another report showed that the neurotoxicity of rotenone on TH-specific, dopaminergic neurons in the mesencephalic culture was ameliorated by CBD (10 µM), with the viability increase to 42% from 29% [33]. The authors suggested that the neuroprotective ability of CBD was deeply related to HO and its products. HO, an enzyme that degrades the heme to biliverdin (BV)/bilirubin (BR), is considered to protect neural cells against excess oxidative stress [60,61]. CBD alone was found to inhibit the activity of HO in a non-competitive manner, which was confirmed by the kinetic analysis on the amount of BV produced. In addition, CBD induced the increase in several mRNA expressions that are associated with stress responses (i.e., HO-1, IL-6, Xbp 1, and CHOP). In contrast, the combined use of CBD and rotenone increased the HO-1 activity by about three-fold compared with the treatment of rotenone only. These results suggested a stress-triggered super-induction of the HO system. The modulatory action of CBD on the HO activity showed similar trends regardless of cell types, indicating that CBD might directly interact with intracellular targets or enzymes rather than being involved in receptor-mediated signaling. On the other hand, CBD, assisted with BV, could almost fully suppress the neurotoxicity of rotenone, as indicated by the viability increase in the neurons and the increase in metabolic HO activity. Mechanisms on the synergistic effect of CBD and BV against oxidative stress remained to be elucidated, but the results showed that BV, as an additive, might aid the CBD-derived stress defense by being supplemented to the cells or directly interfering with the rotenone-induced neurotoxic pathway.

### 2.4. Regulatory Actions on Network Activity and Neuronal Excitability

Anti-epileptic and anti-convulsant properties of CBD have been studied intensively in numerous in vivo and in vitro models, such as PTZ-, pilocarpine-, 3-mercaptopropionic acid-, bicuculline-, picrotoxin-, cocaine-, isoniazid-, and electroshock-induced seizure models [34,35,62]. However, exact mechanisms on the anti-seizure effect of CBD have not been fully elucidated yet, despite the FDA’s approval of Epidiolex^®^, a CBD extract, for epileptic seizures [63]. On the other hand, the modulations of neuronal excitability against dysfunctions of Na^+^ or Ca^2+^ channels and abnormal burst firing of action potential in pathologic conditions, have been suggested for therapeutic targets of anti-epileptic drugs, including CBD [64,65]. 

Ledgerwood et al. reported that CBD had a modulatory ability of basal synaptic activity, inhibiting the spontaneous action-potential (AP) frequency in a concentration-dependent manner, based on the whole-cell patch-clamp studies of primary hippocampal neurons in a current clamp mode [36]. They made several observations, including: (1) PTX completely abolished the AP-inhibitory ability of CBD; (2) among the G_i/o_-coupled GPCRs, CB1 and 5-HT1A receptor, involved in synaptic activity [66,67], were activated indirectly and directly, respectively, by CBD [7]. While CBD (1 µM) induced an 88% decrease in the AP frequency without any alterations of the resting membrane potential, the CBD-induced AP-inhibitory effect decreased to half by an inverse agonist of CB1, but not by an antagonist of the 5-HT1A receptor. These results indicated that CB1 might be indirectly involved in the CBD regulation of synaptic activity via the increase in endocannabinoid levels [53,68], which was known to suppress the neurotransmitter release by the activation of pre-synaptic CB1 [69]. 

Furthermore, CBD in the submicromolar levels (50–5000 nM), a concentration range for plasma CBD in clinical studies, enhanced the AP-firing frequency of primary cortical inhibitory neurons that were induced in vitro by the Dlxi/2b-GFP lentivirus [27]. In a similar fashion, the AP activity of the inhibitory neurons, differentiated from Dravet patient-derived iPSCs, was lower than that from non-patient iPSCs [37], but the AP activity was promoted by CBD (50 nM) (Figure 3g,h). In contrast, CBD repressed the AP firing in the excitatory neurons, differentiated from Dravet syndrome patient-derived iPSCs, suggesting that CBD might rebalance the neuronal excitability in a cell-type dependent manner. However, CBD could not alter the conductance of voltage-gated sodium (Nav) current in both excitatory and inhibitory neurons differentiated from the patient-derived iPSCs, although previous studies showed that the haplo-insufficient mutation of the Nav1.1-encoding SCN1A gene affected the Dravet syndrome [70,71]. 

On the other hand, CBD (10 µM) was reported to reduce the current of peak Nav channel, which was non-synaptically made by the current injection-induced, direct depolarization in a voltage-clamp experiment, to 28% for primary cortical neurons [38]. This result suggested that CBD might act as a Nav channel blocker, which was supported by the observation that the veratridine-induced opening of human recombinant Nav (hNav) 1.1, 1.2, and 1.5 channels was inhibited by CBD, with IC_50_ of about 30 µM, in the hNav-expressing cell lines. However, the blockade of Nav channels by CBD did not seem to be a predominant mechanism in the anti-epileptic properties of CBD, since CBG, which has a comparable ability as a Nav channel blocker to CBD, failed to ameliorate the severity of seizure in the PTZ-induced seizure model. 

Mendis et al. investigated, with the MEA-based analysis, the effects of CBD on neuronal network activity and its mechanisms that were based on N-type voltage-gated calcium (Cav) channels [40]. They reported that CBD induced the synchronous network burst of primary cortical neurons, as inferred from the simultaneous spiking of the multiple neurons with constant period and diminished solitary spiking, compared with the neurons that were not treated with CBD. Furthermore, several network characteristics, calculated with the parameters of firings and bursts, showed the increased regularity of network bursts by CBD. For example, they observed a CBD-induced increase in the ratio of mean firing, which was calculated by dividing the firing rate for the burst period by the mean firing rate for the non-burst period. The result indicated regular and continuous burst firing rather than intermittent firing. CBD also reduced the variation coefficients for various parameters, including network burst duration and rate, overall firing rate, and interval duration between network bursts, supporting that CBD might uniformize the intervals between burst firings or the burst firing rates. In addition, the bioinformatics analysis, including principal component analysis and multi-dimensional scaling, suggested that CBD might interact with the Cav2.2 channel, similarly to omega-conotoxin CVIE, an anti-nociceptive blocker of the Cav2.2 channel. Additionally, CBD (10 µM) fully inhibited the depolarization-induced current of barium (Ba^2+^) flow through the Cav2.2 channel in the Cav2.2-expressed HEK293T cells. Taken together, these results suggested that CBD might directly block the Cav2.2 channel, which participates in pain signaling and has an anti-nociceptive effect. 

## 3. Anti-Gliosis and Anti-Inflammatory Effects of CBD on Primary Glial Cells

Glial cells, such as microglia, astrocytes, and oligodendrocytes in the CNS, are the neural cells that support the structure of the brain as a “glue”, as well as acting as regulators for overall homeostasis and intrinsic defense in the brain [72]. They, when activated under the neuropathological conditions, show the characteristics of self-preservation, which prolong neuroinflammation continuously and chronically through the release of pro-inflammatory molecules or cytokines, stimulate the surrounding glial cells in a paracrine way, and induce additional damages to the neurons [73]. Their cellular responses crosstalk to each other, and therefore, the inhibition of inflammatory reactions of certain glial cells might reduce reactive gliosis of other glial types [74]. This section discusses the effects of CBD on the responses of glia to diverse pathogenic factors—inflammation, glial activation, phagocytosis, [Ca^2+^]_i_ homeostasis, migration, and apoptotic process—and the therapeutic potential of CBD for neurodegenerative diseases by blocking the detrimental, self-accelerating glia-inflammatory cycles (Table 2).

### 3.1. Microglia

Microglia, with the population of up to 12% in the CNS, serve as primary immune cells [83]. Under physiological conditions, microglia in the resting state maintain the homeostasis of the brain and support the neuronal functions by direct contact with the neurons using the few unique processes from their small cell bodies [84]. When pathological events occur, they transform into intrinsic immune effector cells with specialized morphology and then migrate toward the lesion site, remove the damaged cells or pathogens as phagocytes, and promote the neuroinflammatory process by releasing a plethora of cytotoxic agents, including reactive oxygen intermediates (e.g., nitric oxide (NO) and proteases) and inflammatory mediators (e.g., TNF-α, IFNγ, and IL-1β) [85]. In this regard, the functional modulation of reactive microglia, including the suppression of neuroinflammatory ability and improvement of neuro-beneficial ability, has been proposed as one of the therapeutic approaches for neurodegenerative diseases [86].

The anti-inflammation property of CBD on microglia has been supposed in numerous in vivo models of brain disorders [16,17,35,75]. For example, CBD significantly decreased the release of pro-inflammatory cytokines, including TNF-α and IL-1β, from reactive microglia via the activation of the A2A receptor in the TMEV-induced MS model [17]. In addition to the regulatory action on the pro-inflammatory process, CBD increased the expression of anti-inflammatory proteins (e.g., IL-10) and also reduced the oxidative stress by reducing the upregulated mRNA for iNOS in the haloperidol-stimulated tardive dyskinesia disorder [16].

**Figure 4:**
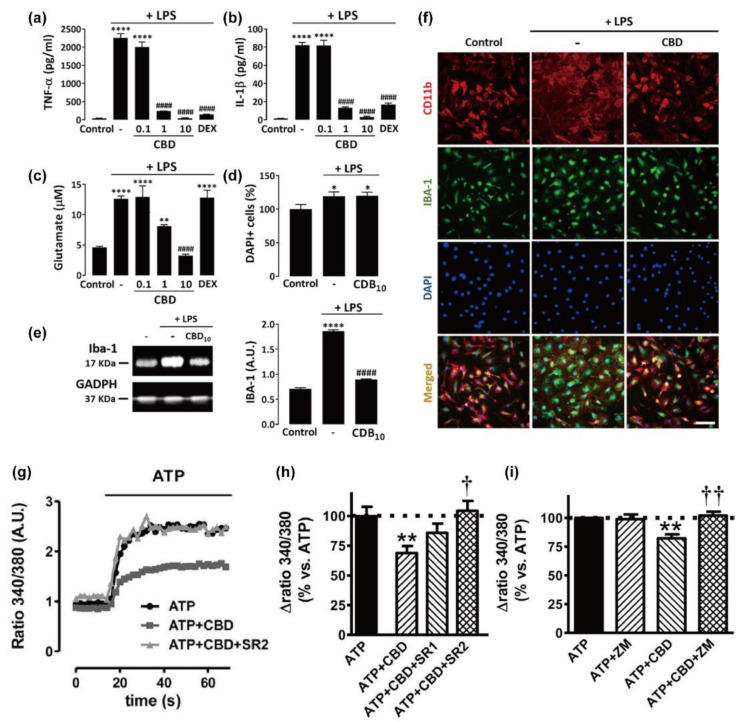
CBD actions on microglia. Anti-inflammatory effects of CBD in the microglial cultures challenged with LPS. (**a**) TNF-α and (**b**) IL1-β releases in the microglial cultures exposed to LPS (10 ng/mL) in the presence or absence of CBD (0.1, 1, and 10 μM) or DEX (2.5 μM). Data are means ± SEM (n = 6), **** *p* < 0.0001 versus controls and ^####^ *p* < 0.0001 versus LPS. (**c**) Extracellular glutamate levels in the microglial cultures exposed to the same treatments as those in (**a**,**b**). Data are means ± SEM (n = 6), **** *p* < 0.0001 versus controls and ** *p* < 0.01, ^####^ *p* < 0.0001 versus LPS. (**d**) DAPI+ cells in the microglial cultures exposed to LPS (10 ng/mL) in the presence or absence of CBD (10 μM). Data are means ± SEM (n = 6), * *p* < 0.0001 versus controls. (**e**) Western blot visualization (**left**) and quantification (**right**) of Iba-1 expression levels in the cultures exposed to the same treatments as those in (**d**). Data are means ± SEM (n = 4), **** *p* < 0.0001 versus controls and ^####^ *p* < 0.0001 versus LPS. (**f**) Visualization of immunofluorescent CD11b (red) and Iba-1 (green) signals and DAPI-counterstained nuclei in the LPS (10 ng/mL)-treated microglial cell cultures in comparison to the control and CBD (10 μM)-treated cultures. Merged images illustrate the global impact of the treatments on microglial cells. Scale bar: 120 μm. (**a**–**f**) Reproduced with permission from [76], Copyright 2019, Wiley Periodicals, Inc. (**g**) Effects of CBD (100 nM) and SR144528 (100 nM, SR2) on ATP-induced increase in the intracellular Ca^2+^ level of primary microglial cells. (**h**,**i**) Implications of (**h**) CB2 and (**i**) A2A receptors in the effect of CBD on ATP-induced increase in intracellular Ca^2+^ level. Statistical analysis was done by one-way ANOVA followed by Student’s *t* test; ** *p* < 0.01 versus ATP, and ^†^ *p* < 0.05 and ^††^ *p* < 0.01 versus ATP + CBD. (**g**–**i**) Adapted with permission from [75], Copyright 2011, The American Society for Pharmacology and Experimental Therapeutics.

On the other hand, there have been limited reports on the CBD effects on inflammatory actions of primary microglia at the cell-culture platform, which is presumably because the amount of primary microglia, obtained from actual animal brains, would not be sufficient enough for the quantitative analysis of protein and gene expression. It was reported that CBD reduced the LPS-induced over-production of NO (360%) to 140% in the primary microglial culture, which was more efficient than other cannabinoids, WIN 55212-2 and JWH-133 (Figure 2) [75]. It was observed that the anti-inflammatory ability of CBD was greater for primary microglia (62% of reduction) than for BV-2 microglia (38% of reduction) under the same conditions. In addition, the LPS-stimulated production and release of pro-inflammatory cytokines (e.g., TNF-α and IL-1β), as well as non-cytokine inflammatory mediators (e.g., glutamate), were significantly reduced by CBD for primary microglia (Figure 4a–c) [76]. CBD (1 and 10 µM) completely inhibited the LPS-triggered release of the inflammatory factors, but CBD of lower concentration (0.1 µM) had no noticeable effect, indicating that the regulatory action of CBD would have a certain threshold concentration. The mechanistic studies with receptor antagonists showed that the CBD effect might not be mediated by PPARγ, GPR55, and CB1, with a marginal contribution of CB2. The anti-oxidizing property of CBD also might play a crucial role in the anti-inflammatory action for microglia. It was observed that CBD completely suppressed the LPS-induced over-production of intracellular ROS by reducing the phosphorylation of p65, a subunit of the NF-κB transcription factor, suggesting that CBD inhibited the NF-κB signaling pathway that was activated by oxidative stress. In addition, CBD abolished the LPS-enhanced production of NADPH, which was required for the ROS production by NADPH oxidase, and it also reduced the LPS-induced increase in the uptake of glucose that is an essential element in the regeneration of NADPH. This result indicated that the inhibitory ability of CBD on the ROS/NF-κB signaling might be mediated by regulating both NADPH production and glucose uptake. Another report showed that CBD diminished the LPS-stimulated ROS level in microglia by more than 50% through its interactions with PPARγ [16]. Taken all together, these reports showed that CBD might block the LPS-stimulated, NADPH/ROS-dependent NF-κB signaling pathway, thus reducing further secretion of pro-inflammatory cytokines in microglia.

In addition to the inflammatory modulation of CBD under neuropathological conditions, CBD was suggested to directly attenuate microglial activation [16,76]. For example, CBD constricted the LPS-stimulated activation of primary microglia and reduced the overexpression of a microglial activation marker, Iba-1, through PPARγ activation (Figure 4d–f). Beneficial effects of CBD on the morphology of activated microglia were also analyzed systematically [16]. The morphology of microglia could be divided into five types: Type I, the cells with two or less cellular processes; Type II, the cells possessing three to five short branches; Type III, the cells with a small cell body and numerous and long processes; Type IV, the cells that have huge soma with retracted and thicker processes; and Type V, the cells that have an amoeboid cell body with numerous short processes and intensely expressing Iba-1 [87]. Among the microglia types, Types III–V are the typical morphologies observed in the microglial activation. The CBD treatment noticeably reduced the microglia of Types IV and V and increased those of Type I in the case of haloperidol-induced mice models for hyperkinetic movement disorder, in which the number of reactive microglia of Types IV and V increased, and those of healthy microglia of Type I was made decreased.

Hassan et al. investigated the effects of CBD on microglial phagocytosis, which is one of the immune responses of microglia [77]. The activity of phagocytosis was calculated by measuring the number of digested, fluorescently labeled beads in the cultured microglial cells. CBD clearly increased the phagocytic activity of primary microglia in a concentration-dependent manner. CBD (10 µM) made a 155% increase in the activity compared with the non-treated condition, while other compounds—phytocannabinoids (CBDA, THC, and cannabidivarin (CBDV) (Figure 2)), endogenous cannabinoids (2-AG and AEA), and synthetic cannabinoids (WIN 55212-2 and JWH-133)—did not enhance microglial phagocytosis regardless of their affinity on the cannabinoid receptors. To identify the regulatory mechanism of CBD on microglial phagocytosis, they investigated the phagocytic ability of BV-2 cells, a mouse microglial cell line, by changing the cellular environments or preventing signaling processes. G_i/o_-linked GPCRs, including CB1 and CB2, were suggested not to be involved in the CBD-based regulation of microglial phagocytosis, based on the following observations: (1) PTX did not affect the CBD-induced phagocytic activity; (2) CB1/CB2-agonistic phytocannabinoids did not increase the phagocytic activity; (3) CBD induced the increase (161%) of phagocytosis for CB1/CB2-deficient BV-2 cells. On the other hand, CBD-induced phagocytic increase was silenced in the extracellular Ca^2+^-absent environment made by EGTA, but not in the intracellular Ca^2+^-deficient condition by BAPTA-AM, implying the potential role of Ca^2+^ signaling in the CBD-based regulation of microglial phagocytosis. In addition, the phagocytic activity, enhanced by CBD, was reduced with various transient receptor potential (TRP) channel blockers—ruthenium red (a non-selective TRP channel blocker), SKF96365 (a TRPC and TRPV blocker), and CPZ or AMG9810 (both TRPV1 antagonists). The phagocytic modulation of CBD was identified to be mediated by the activation of TRPV2 through the quickly stimulated expression of TRPV2 and its promoted translocation to the plasma membrane by CBD. However, the observed different [Ca^2+^]_i_ responses of primary microglia and BV-2 cells, upon CBD treatment, would necessitate further mechanistic studies with a focus on primary microglia: while CBD (10–1000 nM) could not change [Ca^2+^]_i_ in primary microglia [75], CBD (10 µM) increased [Ca^2+^]_i_ by 2.43-fold, and the increased [Ca^2+^]_i_ was sustained even after washing in the case of BV-2 cells [77].

Microglial [Ca^2+^]_i_ was reported to increase upon the quick and sensitive recognition of pathological conditions, leading to inflammatory-related responses [88]. For example, excess ATP, released by damaged neurons and glia, causes the increase in microglial [Ca^2+^]_i_. The ATP level of 400 µM, a similar concentration to its extracellular level that is developed by dying neurons and glia, was applied to the primary microglial cell culture, and the microglia [Ca^2+^]_i_ immediately increased and remained for about 50 s (Figure 4g) [75]. This ATP-induced [Ca^2+^]_i_ increase was significantly reduced to 75% of the initial value by the co-treatment with CBD (0.1 µM). Based on the observation that the antagonists of CB2 or A2A receptor could fully inhibit the role of CBD, the authors suggested that the regulatory actions of CBD on [Ca^2+^]_i_ homeostasis might proceed through both cannabinoid-dependent and -independent pathways (Figure 4h,i). However, they also observed different [Ca^2+^]_i_-responsive profiles in the case of N13 cells: the ATP-induced [Ca^2+^]_i_ increase returned to the base level within 40 s, and the CBD effect on [Ca^2+^]_i_ homeostasis was observed to involve the participation of the A2A receptor but not CB1. 

Under pathological conditions, microglia are changed into amoeba-shaped, mobilizable ones, which migrate to the lesion site and attract immune cells with abundant cytokines or cytotoxic agents. A few studies suggested the potential modulatory ability of CBD on microglial migration. CBD (0.1 µM) promoted the LPS-induced migration of primary microglia with a 2.2-times increase under physiological conditions in the CB1 and CB2-dependent manners [75]. Another study showed that the extracellular addition of CBD alone slightly promoted the microglial migration of BV-2 cells with an EC_50_ of 250 nM, while the addition of 2-AG alone resulted in a more robust increase in the migration with EC_50_ of 25 nM [89]. However, CBD (300 nM) strongly inhibited 2-AG-stimulated microglial migration by more than 90%. The inhibitory action of CBD on 2-AG-induced promotion of microglial migration might be related with GPR18, GPR55, and CB2, because the antagonist of GPR18 and GPR55 (O-1918), and that of CB2 (SR144528) also significantly inhibited the 2-AG-induced migration of BV-2 cells.

The induction of apoptosis of over-activated immune cells is one direction of immunomodulation or immunosuppression. CBD has been suggested to stimulate the apoptosis of immune cells, such as lymphocytes and monocytes [90,91], as well as primary microglia [78]. CBD instigated the apoptotic process of primary murine microglia in the time- and concentration-dependent fashions, leading to the generation of apoptotic characteristics, such as population increase in hypodiploid cells, DNA breaks, fragmented nuclei with condensed chromosomes, and activation of caspase-8 and -9 (initiators of extrinsic and intrinsic apoptotic pathways) [92]. The modulatory action of CBD on apoptosis was suggested to occur through the promotion of lipid-raft coalescence, based on the observation of clustering and brightening up of fluorescently labeled lipids. The CBD-induced apoptotic signals (i.e., morphological changes and caspase expressions) were attenuated by methyl-β-cyclodextrin, a lipid raft disrupter. CBD was also observed to stimulate the microglial expression of ganglioside 1 and caveolin-1, which are components of the lipid raft that were identified to induce the apoptotic signaling in several immune cells, such as lymphocytes, thymocytes, and macrophages [93,94,95].

### 3.2. Astrocytes

An astrocyte is the largest proportional glial type in CNS. They widely provide physiological supportive functions for neurons, such as the provision of nutritional, metabolic, and structural supports, regulation of extracellular ion homeostasis, and assistance to synaptic function with the control of neurotransmitters [72,96]. In neuropathological conditions, astrocytes change to the reactive-state ones, which aggravate neurodegenerative processes through both loss of neuro-beneficial functions and gain of neurotoxic functions. For example, the reactive astrocytes rapidly proliferate and form the glial scars that inhibit axonal regeneration, as well as excessively produce inflammatory cytokines or oxidative stress [97]. 

**Figure 5:**
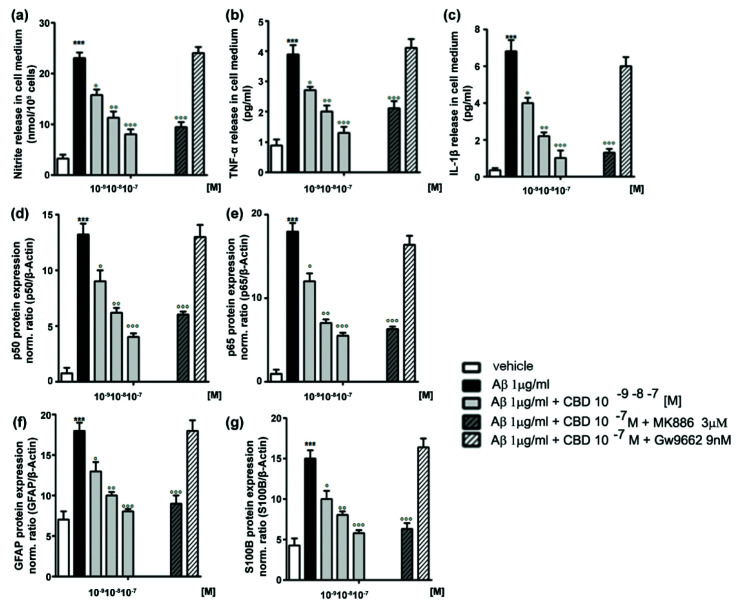
CBD actions on astrocytes. (**a**–**c**) Effects of CBD on the release of inflammatory mediators, (**a**) nitrite, (**b**) TNF-α, and (**c**) IL-1β by the in vitro cultured astrocytes. Aβ-challenged astrocytes (1 mg/mL) were treated with CBD (10^−9^–10^−7^ M) in the presence of PPARα (MK886, 3 mM) or PPARγ (GW9662, 9 nM) antagonist. Measurements were conducted after 24 h. (**d**–**g**) Effects of CBD on the expressions of (**d**) p50, (**e**) p65, (**f**) GFAP, and (**g**) S100B in rat astrocytes. Astrocytes were exposed to the same treatments as those in (**a**–**c**). Western blot analysis was conducted at 24 h after treatments. *** *p* < 0.001 versus control; ° *p* < 0.5, °° *p* < 0.01, and °°° *p* < 0.001 versus Aβ-challenged cells. (**a**–**h**) Reproduced from [73], under the terms of the Creative Commons Attribution License.

CBD has been shown to play a therapeutic role in inhibiting reactive astrogliosis in various in vivo models of brain disorders. For instance, CBD reduced the GFAP-overexpressed reactive astrocytes in the pilocarpine-induced epileptic model through the activation of the PI3K signaling pathway [35]. CBD also suppressed the expression of astrogliosis-marker proteins, GFAP and S100B, via PPARγ activation and NF-κB inhibition in the amyloid β (1–42) peptide (Aβ)-induced AD mouse model [73]. In addition to the in-vivo studies asserting the therapeutic potential of CBD, the regulatory and anti-inflammatory effects of CBD on reactive astrocytes were investigated systematically under the theme of primary astrocytes. CBD inhibited astroglial inflammation, stimulated by Aβ, in a concentration-dependent fashion, by reducing the release of pro-inflammatory molecules and cytokines, such as nitrite, TNF-α, and IL-1β (Figure 5a–c) [73]. CBD also suppressed astrocytic activation, which was indicated by the decrease in S100B and GFAP, in a concentration-dependent manner (Figure 5g). The anti-inflammation and anti-gliosis effects of CBD were involved in the activation of PPARγ, which regulates the expression of genes related with inflammatory responses and lipid/glucose homeostasis [98]. The CBD-induced activation of PPARγ, in Aβ-induced reactive astrocytes, also led to the inhibition of the NF-κB pathway, playing a critical role in astrocytic inflammatory responses [99]. CBD reduced the Aβ-induced overexpression of p50 and p65 proteins, which are subunits of the NF-κB transcription factor, and the effect was abolished by the antagonist of PPARγ (Figure 5e,f).

In addition to the regulation on inflammable gliosis, CBD was reported to universally modulate the entire immune system, including leukocytes, endothelial cells, microglia, and astrocytes. It suppressed the transmigration of leukocytes into the brain by reducing the production of VCAM-1 in endothelial cells and chemokines (e.g., C-C motif ligands, CCL2 and CCL5) in lymphocytes and astrocytes, as well as preventing the microglia activation in the TMEV-induced, demyelinating disease model [17]. In the primary astrocyte culture, CBD inhibited the over-release of CCL2 that attracts monocyte activity, suggesting that the immunomodulatory effect of CBD on astrocytes embraced the suppression of the immune actions of other cells, such as leukocytes. Furthermore, CBD alleviated the TGF-β1/bFGF-induced astrogliosis through the reduction of CSPG production in astrocytes [79]. CSPGs have been known to be upregulated near injury sites, leading to the formation of a glial scar, a barrier, or a blockage of axonal regeneration [100]. The 0.5 μM of Sativex^®^, a 1:1 combination of CBD and THC, inhibited the over-production of CSPGs (e.g., neurocan) from 236% to 95% and normalized the upregulated mRNA expression of core components in the CSPG synthesis (e.g., brevican and XT-1) in the TGF-β1/bFGF-stimulated astroglial culture. These results indicated that CBD provided an environment to permit axonal regeneration and myelin repair by interfering with the accumulation and production of CSPGs. 

The immunomodulatory actions of CBD were investigated in the co-culture systems of several cellular members in CNS, including neurons, microglia, astrocytes, and endothelial cells. CBD (5 μM) attenuated the PrPres toxicity, with viability increase from 37% (without CBD) to 69% (with CBD), in the PrPres-infected neurons co-cultured with N11 cells—non-primary microglial cells, implying that CBD might control the inflammatory responses of reactive microglia triggered by the PrPres deposit, such as the secretion of neurotoxic factors [41]. The anti-inflammatory effect of abnormal CBD (abn-CBD, a regioisomer of CBD, Figure 2) was also investigated in the astrocyte–microglia co-culture [80]. In the co-culture system, the LPS-stimulated increase in pro-inflammatory mediators, such as nitrite and TNF-α, was significantly reduced by abn-CBD in a concentration-dependent manner. Moreover, wound closure after scratch injury was slowed down by abn-CBD (10 µM) at the early phase of culture (0–12 h). This effect was not observed for the isolated astrocyte culture, suggesting that abn-CBD might secondarily inhibit the astrocytic scar formation by its primary effect on microglial activity. 

The exact operational mechanisms of CBD (e.g., inhibition of any astrogliosis-activating actions of microglia or interruption of interactions between microglia and astrocytes) remain unresolved. In this regard, it would be necessary to investigate the complex actions of CBD in the co-culture system of primary cells from the nervous system and get an insight into how CBD regulates the collaborating and stimulating actions in the nervous system. Auzmendi et al. investigated the effects of CBD on the neurovascular unit, which was composed of astrocytes and vascular endothelial cells and regulates the homeostasis of hemodynamics and controls the access of drugs into the brain [82]. They observed that CBD upheld the intracellular level of rhodamine-123 (Rho-123, a probe) in both primary astrocyte–microglia and endothelial H5V cell cultures by inhibiting the leakage of Rho-123 that was caused by hypoxia conditions. Based on the results, they inferred that CBD might inhibit the efflux of Rho-123 by interacting with P-glycoprotein (P-gp) overexpressed in the hypoxia condition. Computational docking experiments showed that CBD might interact with the aromatic residues, such as Phe 728 and Tyr 30, in the α-helix of the P-gp transmembrane domain, via π-stacking, and act as a competitive P-gp inhibitor. Based on the results, they suggested that CBD would resolve the limited entry of anti-epileptic drugs into the brain, which is the characteristic of multidrug-resistant patients.

### 3.3. Oligodendrocytes

The oligodendrocyte is another neuron-supporting glial cell in the CNS. Oligodendrocytes not only produce the axon-wrapping myelin sheath, enabling the rapid and long-distant transfer of action potentials, but also maintain ion homeostasis and supply trophic factors. During development, oligodendrocytes are generated from oligodendrocyte progenitor cells (OPCs), but a large portion of OPCs remains undifferentiated and immature as a potential repository for remyelination [101]. Oligodendroglial dysfunctions (e.g., dysmyelination/demyelination), potentially leading to severe neurodegeneration, have been characteristically observed in several neurodegenerative disorders, such as AD, ALS, and MS [102,103]. Although therapeutic studies, targeting oligodendrocytes, have continuously been conducted from the perspective of remyelination strategies that include oliogodendroglial differentiation, maturation, and remyelination, little is known about the effects of phytocannabinoids, including CBD, on the behaviors of oligodendrocyte lineages [104]. 

CBD (>0.1 µM) increased the death rate of primary oilgodendrocytes, while primary cortical neurons did not show any cytotoxic results with 10 µM of CBD [42,78]. In addition, the apoptosis rate of primary microglia was not increased by CBD (<4 µM), suggesting that oligodendrocytes were much more sensitive to CBD than other primary neural cells. The oligodendro-toxicity of CBD might be related to the dysregulation of [Ca^2+^]_i_ homeostasis by CBD but not to receptor-mediated pathways (e.g., CB1/2, TRPV1, PPARγ, and A2A receptor), based on the observation that the cytotoxicity of CBD was reduced only in the depletion of extracellular Ca^2+^. In a similar trend to the aforementioned induction of neuronal [Ca^2+^]_i_ increase by CBD [25,28,29,30], CBD was also reported to increase [Ca^2+^]_i_ by 43% in the non-specific cortical glial cells. The [Ca^2+^]_i_ increase in the glia was observed to be made through L-type VGCC and intracellular Ca^2+^ stores, but it was negatively controlled by CB1 and TRPV. In addition, the proportion of CBD-reacting cells was higher for glia than for neurons [28,30]. Specifically, CBD induced the [Ca^2+^]_i_ increase in primary oligodendrocytes in a concentration-dependent manner, and the increase was blocked partially by the elimination of extracellular Ca^2+^ or mitochondrial impairment, not by the blockades of CB1/2, TRPV1, L-type VGCC, ryanodine, and IP_3_ receptors [42]. The CBD-induced apoptotic process was also mediated by the extrinsic and intrinsic apoptotic cascades, related with caspase-8 and -9, and by the caspase-independent necrotic pathways related with PAPR-1, which is a nuclear enzyme involved in the AIF-induced cell death [105]. In addition, it was found that CBD (0.1, 1, and 10 µM) increased the intracellular concentration of ROS, and the oligodendro-toxicity of CBD (1 µM) was attenuated by Trolox, a ROS scavenger, with 35.4% inhibition of cell death. CBD (1 µM) induced the continuous hyperpolarization of mitochondrial membrane potential (MMP), while CBD (10 µM) caused the hyperpolarization, at the initial point, and subsequent depolarization of MMP, implying that MMP might be directly interfered by the lipophilic property of CBD. Based on the results, they suggested that the CBD-induced ROS over-production and MMP disruption might crosstalk with the [Ca^2+^]_i_ increase in oligodendrocytes.

**Figure 6:**
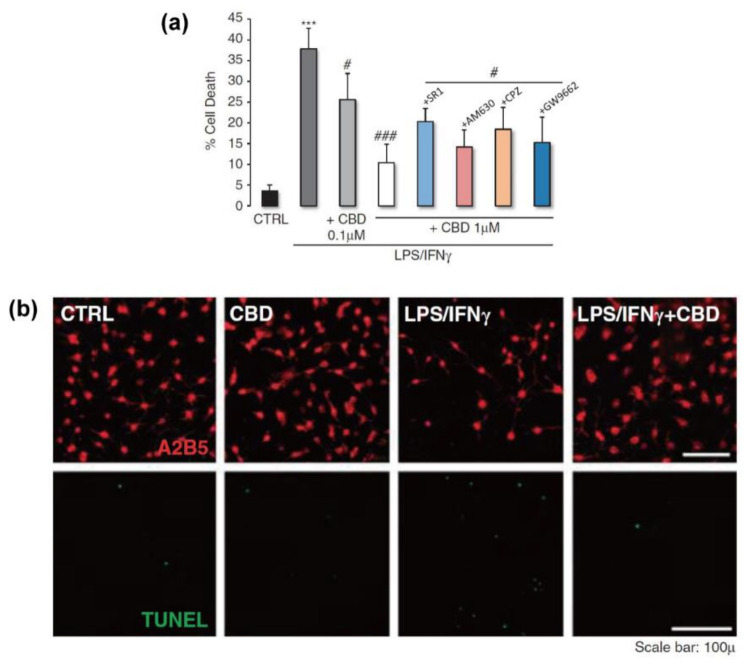
CBD actions on OPCs. (**a**) Protective effect of CBD on LPS/IFNγ-induced cytotoxicity in OPCs. CB1, CB2, TRPV1, or PPARγ antagonists (SR1, AM639, CPZ: 1 mM and GW9662: 50 nM) were administered 30 min before the stimulus to explore the effect of various receptors. The statistical analysis was performed using Kruskal–Wallis ANOVA followed by Mann–Whitney U test; *** *p* ≤ 0.001 versus non-treated cells, ^#^ *p* ≤ 0.05 and ^###^ *p* ≤ 0.001 versus cells exposed to LPS/IFNγ alone. (**b**) A2B5 and TUNEL staining of OPCs exposed to LPS/IFNγ for 24 h in the presence or absence of CBD (1 µM). (**a**,**b**) Reproduced from [81], under the terms of the Creative Commons Attribution-Noncommercial-NoDerivs 3.0 Unported (CC BY-NC-ND 3.0) License. Copyright 2012, The authors, published by Springer Nature.

Unlike the results for oligodendrocytes, CBD (1 µM) did not induce any cytotoxicity, [Ca^2+^]_i_ increase, nor alterations of the cell cycle and proliferation rate for primary OPCs [81]. Moreover, CBD could protect OPCs against the LPS/IFNγ-induced inflammation: the cell death of 37.8%, by LPS/IFNγ, was significantly reduced to 10.4% by the co-treatment with CBD (1 μM) (Figure 6). CBD also reduced the overexpression of caspase-3 protein, an apoptotic effector, and the number of DNA-fragmented cells under the LPS/IFNγ-induced inflammation. The anti-inflammatory effect of CBD on OPCs seemed to be operating through receptor-independent mechanisms, because no alterations were observed in the protective effect of CBD, when the antagonists of the receptors, including TRPV1, PPARγ, and CB1/2, were co-administered with CBD. CBD also rescued OPCs from H_2_O_2_-induced oxidative stress, with a decrease in cell death to 39% from 85%, by partially suppressing ROS production. In addition to the protection against inflammation and oxidative stress, CBD attenuated the cytotoxicity of tunicamycin-induced ER stress in OPCs with a decrease in cell death from 46% to 20%, by reducing phosphorylated eiF2α, which is an initiator of ER stress-derived apoptosis. In the case of LPS/IFNγ-stimulated inflammatory condition, CBD also increased the amount of phosphorylated PKR, an ER transmembrane protein factor, and also phosphorylated eiF2α, which is formed by PKR in response to ER stress that initiates the apoptotic pathway [106]. These results suggested that the OPC-protective properties of CBD against ER damage and its associated inflammation were mediated by suppressing the ER stress-induced apoptotic process, including the PKR–eiF2α pathway, and modulating the expressions of pro-apoptotic and anti-apoptotic factors (e.g., caspase-12, Bcl-2, and Bax). 

## 4. Regeneration from Neural Stem Cells

Neural stem cells (NSCs), having self-renewing and multipotent capacities in the specific lineages, including neurons, astrocytes, and oligodendrocytes, have been proposed as one of the potential treatment targets for neurological diseases [107,108,109]. The NSC therapies, such as regenerative therapy and cell-replacement therapy, could be made more effective with the assistance from pharmacological agents that have the following properties: (1) aiding the survival of transplanted cells or newly born cells; (2) boosting neurogenic activity to promote the proliferation of neural progenitor cells (NPCs); and (3) inducing the differentiation/maturation into desired lineages, such as neurogenesis [107,110]. A few studies have suggested that CBD modulates the NSC activities, including its survival, proliferation, and differentiation, and it has the therapeutic effects on neurological diseases, such as anxiety disorder, through the regulation of neurogenesis [110]. In this section, we discuss both in vivo and in vitro studies (Table 3) that have showed the potential of CBD in the regulation of NSC neurogenesis.

Several in vivo studies under physiological conditions have been conducted to uncover the effect of CBD on the NPC proliferation and adult neurogenesis in the NSC-enriched niches, including the subventricular zone (SVZ) and subgranular zone (SGZ) [108]. Campos et al. observed that the consecutive intraperitoneal injection of CBD (30 mg/kg) to mice for 14 days significantly increased several types of cells in the SGZ of hippocampi: newborn and survived proliferating cells (labeled, at the beginning of the CBD injection, by BrdU as a marker of the proliferating cells in the S-phase); NPCs or immature neurons (DCX-expressing cells); newborn and matured neurons (BrdU-positive and NeuN-expressing cells) [113]. Shiavo et al. also reported that the repeated daily injection of low-dose CBD (3 mg/kg) to mice for 15 days greatly increased the number of Ki-67 (a marker protein for cell proliferation)-expressing or BrdU-positive cells and DCX-expressing cells in both SVZ and SGZ [115]. However, CBD at a higher dose (30 mg/kg) caused the reduction in the cell numbers, implying that the CBD activity on proliferation and neurogenesis would be dose-dependent, and the effective dose range for the CBD action might be narrow.

Wolf et al. studied the influence of a continuous CBD-enriched diet on cell proliferation and adult neurogenesis in dentate gyrus (DG) of mice for 6 weeks [116]. After 6 weeks of CBD treatment, the proliferating cells in the S-phase were labeled with BrdU via intraperitoneal injection. Cell proliferation was observed after 24 h of BrdU injection, and cell survival and differentiation were characterized after 4 weeks of BrdU injection. CBD reduced the number of the BrdU-positive, proliferating cells but significantly increased the number of the survived, BrdU-positive cells as well as the survived and differentiated cells (BrdU-positive and NeuN-expressing cells). This result suggested that CBD might improve cell proliferation, survival, and net neurogenesis in the DG. The CBD effect on cell survival in the DG was completely abrogated in the CB1-deficient mice. They also investigated the effect of CBD on each development stage in the differentiation process by classifying the types of differentiating cells in the DG into type 1/2a-progenitor (1/2a-P) cells, type 2b-progenitor (2b-P) cells, type 3-progenitor (3-P) cells, and postmitotic neurons (PMNs): the 1/2a-P cells express nestin but not DCX; the 2b-P cells express both nestin and DCX; the 3-P cells expressed DCX but not nestin; the PMNs expressed NeuN. Whereas CBD did not alter the number of 1/2a-P and 3-P cells, compared with the non-treated group, it significantly decreased that of 2b-P cells and increased that of PMNs, implying that CBD might induce the passing of developmental stages for 2b-P cells but ultimately make more NPCs mature. 

CBD was reported to exhibit the protective effects against the impairment of adult neurogenesis in pathological conditions, such as CUS-induced anxiety and Aβ-induced neuroinflammation. For example, the CUS-derived anxiety of mice caused the reduction in the number of BrdU-positive and DCX-expressing cells in SGZ, but the daily injection of CBD, after CUS stimulation, completely abolished the CUS-induced reduction of these cells [113]. The elevated plus-maze and novelty-suppressed feeding tests also showed that CBD inhibited the CUS-induced, anxiety-like behaviors. The CBD action in the attenuation of CUS-induced anxiogenic behaviors was suppressed by treating thymidine kinase-expressing transgenic mice with GCV, which blocked the NPC proliferation, suggesting that the protective effects of CBD on proliferation in the NSC-enriched niche might be involved in the anxiolytic effect of CBD. It was also reported that CBD restored the CUS-induced reduction in the number of differentiating and migrating cells (DCX-expressing cells in between SGZ and granular zone (GZ)) and newborn and matured neurons (BrdU-positive and NeuN-expressing cells) [117]. Furthermore, CBD thoroughly rescued the mice from CUS-derived dendritic impairment in the DG by accelerating dendritic remodeling including increases in spine density in secondary or tertiary branches, the size of dendritic trees, and the total number of branches. In the AD rat model induced by Aβ injection, the continuous administration of CBD (10 μg/mL) for 15 days increased the number of DCX-expressing cells as well as the expression of calbindin (a marker protein of mature neurons), suggesting that CBD stimulated neurogenesis in the DG [73].

On the other hand, there have been few reports on in vitro studies on CBD effects on NSCs. Mazzon et al. reported that CBD promoted the expression of the genes related to the neural differentiation of hGMSCs, which could differentiate into osteogenic, adipogenic, or neurogenic lineage [111]. The 24 h CBD treatment (5 μM) did not cause any cytotoxicity on the viability and morphology of hGMSCs, but CBD above 10 μM led to cell death and abnormal cell morphology. Therefore, they quantitatively compared the transcriptomes of CBD (5 μM)-treated and DMSO (0.1%)-treated hGMSCs (the control). The CBD-treated hGMSCs significantly upregulated the expression of several genes associated with neural differentiation (e.g., BCL9, BMP4, C11orf70, C2orf16, CBX8, CENPH, CEP78, DPCR1, SEMA6D, GRIK2, and NES), nervous system development (e.g., CHRM2, FOS, and BTD), and axonogenesis/axon developement (e.g., NEFH and NEFM). They also found that the proportion of NEFM- or CHRM2-expressed hGMSCs markedly increased in the case of continuous CBD treatment for 4 days, but not for 2-day treatment, while that of TP53 (a gene known to negatively regulate neurogenesis of NSCs [118])-expressed hGMSCs decreased more for 4-day treatment than for a 2-day one. CBD also led to the downregulation of gene expressions related to differentiation into other lineages, including oligodendrocyte development (e.g., GALC and CSAD), angiogenesis (e.g., PDGFC), and osteogenesis (e.g., CBFB). 

**Figure 7:**
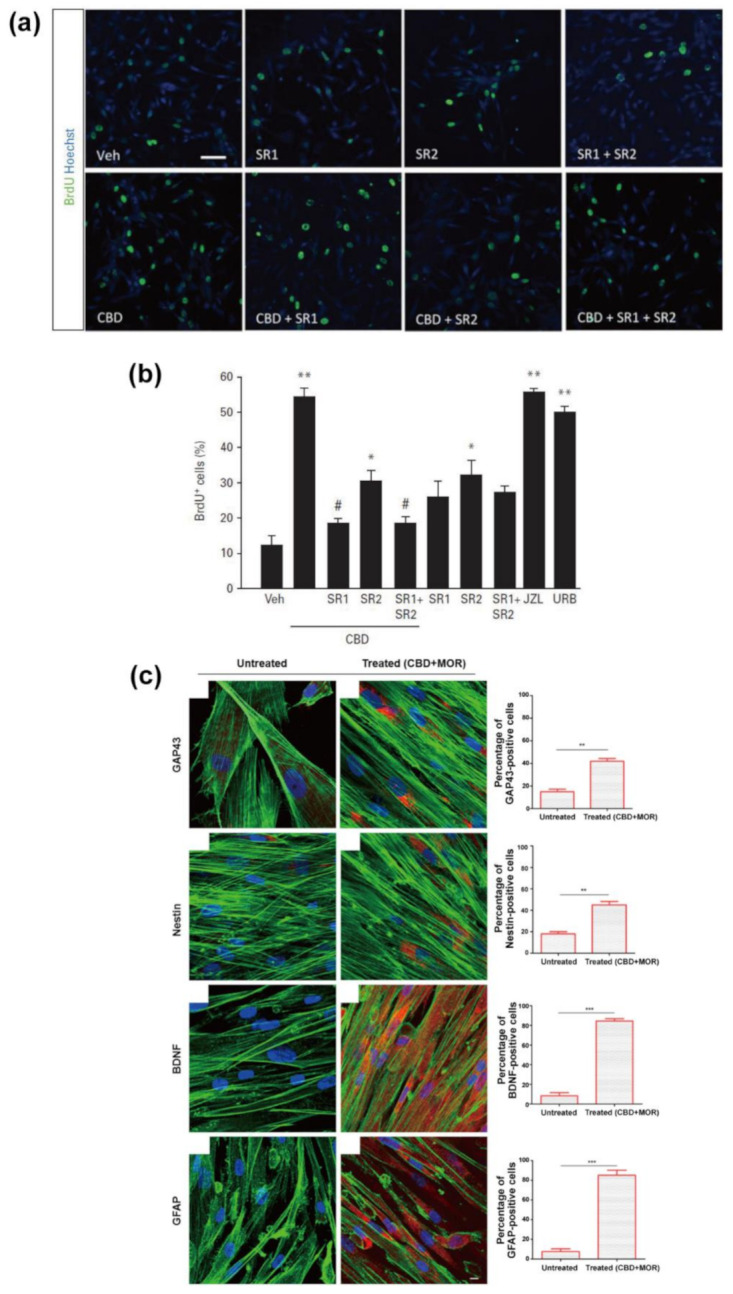
CBD actions on stem cells. Proliferative effects of CBD (100 nM) on neural progenitor cells were determined by (**a**) the analysis of immunofluorescence images and (**b**) the quantification of BrdU-positive cells, in the presence of SR141716 (SR1) and SR144528 (SR2), either alone or together. Analysis of variance followed by Duncan’s post-hoc test, * *p* < 0.05, ** *p* < 0.01 versus the respective vehicle-treated cells. ^#^ *p* < 0.05 versus the respective CBD-treated cells. (**a**,**b**) Reproduced with permission from [113]. Copyright 2013, Oxford University Press. (**c**) Immunofluorescence analysis with GAP43, NES, BDNF, and GFAP for the control hPDLSCs and the CBD/MOR-treated hPDLSCs. Histograms represent the percentage of positive cells for the specific markers. ** *p* < 0.01, *** *p* < 0.001 significant difference of hPDLSCs treated with CBD and MOR compared to non-treated cells. Reproduced from [112], under the terms of the CC-BY Creative Commons Attribution 4.0 International license (https://creativecommons.org/licenses/by/4.0/, accessed on: 10 August 2021).

Mazzon et al. also studied the co-effect of CBD with moringin (4-(α-L-rhamnosyloxy)-benzyl isothiocyanate, MOR), which is one of the phytocompounds from *Moringa oleifera* seed, on the survival and neuronal differentiation of hPLMSCs [112]. The combined treatment of CBD (0.5 μM) and MOR (0.5 μM) increased the number of viable cells by 20% and induced the alteration of cellular morphology into a more roundish shape, rather than spindle shape, by the rearrangement of F-actin. In addition, the 48 h CBD/MOR co-treatment significantly increased the proportion of the cells that expressed certain proteins: a 40% increase for nestin and GAP43 (a specific protein of neurogenic lineage), and an 80% increase for BDNF (a brain-derived neurotrophic factor that has been known to support the survival of newborn neurons [119]) and GFAP (an early marker of neurogenic lineage) (Figure 7c). The transcriptome analysis showed that the co-treatment statistically induced the regulation of 195 genes associated with the inhibition of apoptosis and the PI3K/Akt/mTOR pathway, which has been known to control the neuronal survival [120,121]. CBD with MOR might promote trans-differentiation into neurogenic lineage and the stem-cell survival, and the co-treatment would have therapeutic advantages in stem-cell therapy for neurological diseases [107].

Campos et al. studied the effects of CBD on the proliferation and cell cycle of HiB5 hippocampal progenitor cell line (Figure 7a,b) [113]. The 16 h CBD administration (100 nM) increased the population of BrdU-positive cells to 54% from 12% for the vehicle (0.1% DMSO) control. In particular, CBD was found to induce the progression of the G1-S phase in the cell cycle. The co-administration of the antagonist of CB1 or CB2 (SR141716 or SR144528, respectively) with CBD inhibited the promoting ability of CBD on the proliferation (percentage decrease in BrdU-positive cells to 19% or 31%, respectively). In addition, the intracellular AEA depletion by FAAH overexpression, which was induced by transfecting the pCIG2-FAAH-expressing vector, completely abrogated the CBD-induced increase in BrdU-positive cells. These results suggested that the improvement effect of CBD on the proliferation of hippocampal progenitor cells might be related to the endocannabinoid system through the indirect receptor activation, for example by alteration of endocannabinoids levels.

On the other hand, Bekman et al. reported that CBD neither promoted nor inhibited the neuronal differentiation and maturation of the NPCs that were differentiated from ihPSCs [114]. The CBD treatment (10 μM) led to extensive cell death within 2–3 days, and CBD of 1 μM reduced the cell density on the 11th exposure day. However, the CBD (1 μM)-treated cells in differentiation-induced media, after 11 days, had typical NPC morphology (e.g., neural rosettes [122]) and expressed the comparable extent of mRNAs for MAP2 (a marker of neuronal differentiation) and PAX6 (an NPC marker) to the vehicle (0.01% EtOH) control. In addition, the CBD-treated neurons in differentiation/maturation-induced media, after 37 days, exhibited the functional Ca^2+^ signaling activity. For example, all the CBD-treated cells increased [Ca^2+^]_i_ immediately as a response to the KCl stimulation, which induces the Ca^2+^ influx into the cytosol via VGCCs [123], with the averaged [Ca^2+^]_i_ increase slightly less than the vehicle control. Furthermore, only 8.7% of the CBD-treated cells reacted to the histamine stimulation, which induces the [Ca^2+^]_i_ increase through the histamine receptors that are known to be expressed mostly in immature neurons. The 37-day CBD treatment increased the expression levels of CNR2, encoding the CB2, and GAD67 (a marker of GABAergic neuron), and it decreased that of VGLUT1 (a marker of glutamatergic neurons).

## 5. Perspective and Outlook

CBD is not just one of the 100+ compounds extracted from the *Cannabis* genus and identified, but one of the two main phytocannabinoids, CBD and THC, which have unceasingly attracted profound attention and interest from researchers as well as the general public from the aspect of their potential health benefits. Compared with THC that is psychoactive, non-psychoactive CBD has been on the market in various forms (e.g., oil, gels, gummies) for cosmetic and “allegedly nutraceutical” products in some countries. CBD has been claimed to have many health benefits, such as pain and anxiety relief, symptom alleviation for epilepsy and cancer, management of neurodegenerative diseases, and acne treatment, but many of them have not so far had solid scientific data for supporting the suppositional benefits [10]. The recent FDA’s approval of a CBD extract (brand name: Epidiolex^®^), as a prescription medicine for the treatment of seizures associated with Lennox-Gastaut syndrome, Dravet syndrome, or tuberous sclerosis complex in patients 1 year of age and older, has boosted the interest in CBD as a potential therapeutic candidate for other diseases, including neurodegenerative diseases. Moreover, diverse drugs containing CBD in various formulations have been proposed or are actively under development, such as Sativex^®^, an oromucosal spray composed of CBD and THC, for the treatment of spasticity and pain related to MS [79,124], PTL101, a CBD-embedded oral gelatin matrix pellet for the adjunctive treatment of pediatric epilepsy [125,126], and Zygel^TM^ (ZYN002), a CBD-formulated transdermal gel for the treatment of Fragile X syndrome [127,128].

This review has focused on the biochemical actions of CBD in neural cells at the cell-culture level in order to provide an overview of the research efforts for a molecular level, mechanistic understanding of how CBD affects the neural-cell behaviors under normal and pathological conditions (Table 4). Discussion has been made, separately, for each cell type—neurons, microglia, astrocytes, oligodendrocytes, and neural stem cells. As for primary neurons, CBD has been reported to exhibit protective ability, assisting in the prevention of massive neuronal death, by being involved in a series of cell-death processes and the normalization of neuronal functions. CBD also shows therapeutic potential for neurological diseases by targeting glial cells, as indicated by its inhibitory action against gliosis/inflammation that might accelerate neuronal degeneration and exacerbate the progression of neurological diseases, in addition to its protective action for neuro-beneficial cells (e.g., oligodendrocyte progenitor cells) against pathogens. Moreover, CBD has been found to support the survival of newborn neural cells, intensify neural progenitor cells, and expedite neuronal differentiation, showing the therapeutic possibility of CBD in NSC therapy. 

However, the limited number of research articles has so far been published on in-vitro culture-based studies of CBD actions on neurological diseases and others, presumably because of the legal scheduling of *Cannabis* and the low amount of pure CBD extracted from *Cannabis*. The interdisciplinary nature of CBD studies also might deter the in-depth fundamental research on CBD effects on neural cells in the brain, because it requires the synergistic contributions from neuroscience, medicinal chemistry, biochemistry, biology, and bioinformatics to name a few.

CBD production has relied mainly, if not solely, on the natural source, *Cannabis* plant, which would defer the mass production of CBD at its highly pure form. Mass production will be beneficial to the fundamental study of the CBD action not to mention for its medical use at the market. Therefore, it is recommended to develop alternative methods for the mass production of pure CBD. Total synthesis would be one of the strategic options [129]. Not only the approach of total synthesis is seamlessly coupled with the existing mass-production methods, but also it would provide methodological ways to the structural analogs of CBD, which would give higher efficacy than CBD for a given disease state. The structural analogs also might be useful for mechanistic elucidation of CBD actions to neural cells. Another alternative would be metabolic engineering [130], which could be combined with synthetic organic methods for increasing the entities in the chemical compound space. Recently developed gene-editing techniques, such as CRISPR, would be an advanced option for further increasing the production yield in the biosynthetic approach.

It is anticipated that detailed understanding, at the fundamental level, with solid experimental data, in the combination with the results from (pre)clinical studies, would solidify the path to the medical use of CBD. For example, the biological actions are complex and intertwined, and its network is highly interconnected in the many-to-many mode, although this review has primarily dealt with the CBD actions on each cell type in the neural cells separately. Further studies on cell-to-cell interactions induced by CBD would give more mechanistic information at the molecular level. Contributions from other domains also could be made, such as the fabrication of artificial tissues or organoids in biological and biomedical engineering. The cellular construct is innately three-dimensional (3D), but most of the previous studies discussed in this review have heavily relied on the 2D culture, which might not faithfully recapitulate the cellular states in the brain in vivo from the chemical, biological, and mechanical viewpoints [131,132,133].

## Figures and Tables

**Table 1:** CBD actions on primary neurons.

Cell Type/Source	DIV	Toxicant	CBD Concentration [μM]	CBD Exposure	CBD Effects	Related Molecular Targets	Ref.
cortical neurons (Wistar rat pups, E17)	7–18	glutamate; *tert*-butyl hydroperoxide	1/3.16/10/31.6	18–20 h	neuroprotection against NMDAR-mediated and AMPA/kainate receptor-mediated glutamate toxicity, ROS-induced toxicity, and the glutamate-induced toxicity	(not CB1/2)	[23]
cerebellar granular neurons (Wistar rats, 6–8 days)	6–7	H_2_O_2_; rotenone	2.5/5/10	24/48 h	neuroprotection against ROS-induced toxicity and mitochondrial dysfunction	(not CB1/2 and 5-HT1A receptor)	[26]
hippocampal neurons (Sprague–Dawley rat pups, E18)	1	H_2_O_2_	0.1/1/3/5/10/15/30/50/100	24 h	neuroprotection against H_2_O_2_-induced toxicity		[24]
hippocampal neurons (Sprague–Dawley rats, 1–3 days)	5–10		1/10	0	regulation of [Ca^2+^]_i_ homeostasis	Ca^2+^ channels (including L-type VGCC), CB1, and TRPV1	[28]
hippocampal neurons (Lister-hooded rats, 1–3 days)	5–10		1	0	regulation of [Ca^2+^]_i_ homeostasis		[29]
hippocampal neurons (Lister-hooded rats, 1-3 days)	5–10		1	0	regulation of [Ca^2+^]_i_ homeostasis	CB1, TRPV1, and PLC; (not FAAH, MAGL, and G_i/o_-linked GPCR)	[30]
hippocampal neurons (Lister-hooded rats, 1–3 days)	5–10	4-AP; oligomycin; FCCP	0.1/1	0/overnight	regulation of [Ca^2+^]_i_ homeostasis; neuroprotection against oligomycin and FCCP	mNCX; (not mPTP, and ryanodine and IP_3_ receptors)	[25]
cortical neurons (C57BL/6J mouse pups, E13)	2–3		1/10/20	0	regulation of [Ca^2+^]_i_ homeostasis	(not G_i/o_-linked GPCRs and CB1/2)	[31]
mesencephalic neurons (OF1/SPF mouse pups, E14)	11	MPP	0.01/0.1/1/10	48 h	neuroprotection against MPP-induced toxicity		[32]
mesencephalic neurons (OF1/SPF mouse pups, E14)	12	rotenone	10	48 h	neuroprotection against rotenone-induced toxicity		[33]
hippocampal neurons (Sprague–Dawley rat pups, E18)	21	PTZ	1.89	1 h	reduction of PTZ-induced increase in methionine level		[34]
hippocampal neurons (C57BL/6 mice, 1 day)	10–12	glutamate	0.1/1/10	4 h	neuroprotection against glutamate-induced toxicity	PI3Kγ	[35]
hippocampal neurons (Sprague–Dawley rats, 1–2 days)	13–16		0.1/1/10	5 min	regulation of synaptic activity	G_i/o_-linked GPCR and CB1; (not 5-HT1A receptor)	[36]
cortical neurons (Sprague–Dawley rat pups, E18)	13–14		0.005/0.015/0.05/0.15/0.5/1.5/5/15	5–6 min/48 h	regulation of neuronal activities ([Ca^2+^]_i_ and action potential)		[37]
telencephalic inhibitory and excitatory neurons (Dravet syndrome patients-derived iPSCs)			0.05	5–6 min	regulation of action potential activity		[37]
cortical neurons (NIHS mouse pups, E13–15)	4–11		10	20 min	regulation of Nav channel		[38]
cortical neurons (C57BL/6 mice, 0–2 days)	14–21		10	10 min	regulation of network activity	Cav2.2 channel	[39]
mesencephalic neurons (OF1/SPF mouse pups, E14)	12		0.1/1/10	48 h	neurotoxicity		[40]
cortical neurons (C57BL/6 mouse pups, E14)	6–8	PrPres	1/5	48 h	neuroprotection against PrPres		[41]
cortical neurons (Sprague–Dawley rat pups, E18)	8		0.1/1/10	20–30 min	neurotoxicity		[42]

**Table 2:** CBD actions on primary glia.

Cell Type/Source	DIV	Toxicant	CBD Concentration [μM]	CBD Exposure	CBD Effects	Related Molecular Targets	Ref.
microglia (cortices of neonatal rats)	1	ATP; LPS	0.001–1	0/24 h	anti-inflammatory effect; promotion of migration; [Ca^2+^]_i_ regulation	CB1/2 and A2A receptor	[75]
microglia (brains of C57BL/6J mice)		LPS	0.1/1/10	24 h/30 min	anti-inflammatory effect; astrogliosis inhibition	CB2; (not CB1, GPR55, and PPARγ)	[76]
microglia (brains of C57BL/6J mice)	14–16	LPS	10	4 h	anti-inflammatory effect; inhibition of microglial activation	PPARγ	[16]
microglia (brains and spinal cords of C57BL/6 mice, 8–10 weeks)	4		10	24 h	promotion of phagocytosis	TRP channels (TRPV1, TRPV2, and TRPC); (not G_i/o_-linked GPCR and CB1/2)	[77]
primary microglial cells (forebrain of newborn BALB/c mice)			1/4/8/16/24	1-24 h	induction of microglial apoptosis	(not CB1/2, TPRV1, and GPR55)	[78]
astrocytes (cortices of Sprague–Dawley rats, 2 days)		Aβ1-42	0.001/0.01/0.1	24 h	anti-inflammatory effect; astrogliosis inhibition	PPARγ; (not PPARα)	[73]
astrocytes (Wistar rats, 0–2 days)		IL-1β + TNF-α	1/5	6 h	immunosuppression	A2A receptor	[17]
astrocytes (Wistar rats, 0-2 days)	4	TGF-1β + bFGF	Sativex^®^ (0.1/0.5/1)	24/48/72 h	astrogliosis inhibition		[79]
astrocytes (brains of C57BL/6 mice, 0–1 day)	1	LPS; scratch	1/10	30 min/72 h	anti-inflammatory effect; astrogliosis inhibition	(not CB2)	[80]
oligodendrocytes (optic nerves of Sprague–Dawley rats, 12 days)	1		0.1/1/10	0/20–30 min	oligodendro-toxicity; [Ca^2+^]_i_ regulation	(not CB1/2, TPRV1, PPARγ, A2A, ryanodine and IP_3_ receptors, and L-type VGCC)	[42]
OPCs (brains of Wistar rats, 0–2 days)	3	H_2_O_2_; tunicamycin; LPS/IFNγ	0.1/1/2.5/5	2/24/48 h	oligodendro-protection	(not CB1/2, TPRV1, and PPARγ)	[81]
astrocytes and microglia (brains of C57BL/6 mice, 0–1 day)	1	LPS; scratch	1/10	30 min, 72 h	anti-inflammatory effect; astrogliosis inhibition	(not CB2)	[80]
astrocytes and microglia (cortices of Wistar rats, 3–5 days)		CoCl_2_	5/50/100	30 min	regulation of hemodynamics	P-gp	[82]
mixed glial cells (hippocampi of Lister-hooded rats, 1–3 days)	5–10		1	0	[Ca^2+^]_i_ regulation		[29]
mixed glial cells (hippocampi of Lister-hooded rats, 1–3 days)	5–10		1	0	[Ca^2+^]_i_ regulation	CB1, TRPV1, and PLC; (not FAAH, MAGL, and G_i/o_-linked GPCR)	[30]
mixed glial cells (hippocampi of Sprague–Dawley rats, 1–3 days)	5–10		1/10	0	[Ca^2+^]_i_ regulation	Ca^2+^ channels (including L-type VGCC), CB1, and TRPV1	[28]
mixed glial cells (hippocampi of Lister-hooded rats, 1–3 days)	5–10	4-AP; H_2_O_2_; oligomycin	1	0	[Ca^2+^]_i_ regulation	mNCX; (not mPTP, and ryanodine and IP_3_ receptors)	[25]

**Table 3:** CBD actions on stem cells.

Cell Type	Cell Source	DIV	CBD Concentration [μM]	CBD Exposure	CBD Effects	Related Molecular Targets	Ref.
MSCs	gingival tissues of healthy adult patients	2nd passage	5/10/25	24/48/96 h	promotion of differentiation toward neural progenitor cells		[111]
MSCs	periodontal ligament tissues of healthy adult patients	1st passage	CBD + MOR (0.5 each)	24/48/72 h	promotion of cell survival and neuronal differentiation		[112]
NPCs	HiB5 hippocampal progenitor cell line		50/100/250/500	16/48 h	promotion of cell survival and proliferation; and modulation of cell cycle	CB1/2; (not 5-HT1A receptor)	[113]
PSCs	human-induced PSC lines (iPSC6.2 and F002.1A.13)	19	1/10	11/37 days	effect on differentiation/maturation of neural progenitor cells.		[114]

**Table 4:** Summary for reported biochemical actions of CBD in neural cells.

Cell Type		Biochemical Actions	Ref.
neurons		anti-oxidation	[21,23,24,25]
		[Ca^2+^]_i_ homeostasis	[25,28,29,30,31]
		protection against mitochondrial dysfunction	[26,32,33]
		regulation of neural-network activity	[36,37,38,39]
glia	microglia	anti-inflammation	[16,17,35,75,76,79,80]
		regulation of microglial activation	[16,17,35,76,79,80]
		promotion of phagocytosis	[77]
		[Ca^2+^]_i_ homeostasis	[25,28,29,30,75,77]
		microglial migration	[41,75,89]
		induction of microglial apoptosis	[78]
		regulation of haemodynamics	[82]
	astrocytes	anti-inflammation	[73,79,80]
		regulation of astroglial activation	[35,73,79,80]
		immunosuppression	[17]
		regulation of haemodynamics	[82]
		[Ca^2+^]_i_ homeostasis	[25,28,29,30]
	oligodendrocytes	oligodendro-protection	[81]
		oligodendro-toxicity	[42]
		[Ca^2+^]_i_ homeostasis	[25,28,29,30,42,81]
neural stem cells		promotion of proliferation	[111,113,115,116,117]
		support of survival	[112,113,115,116,117]
		promotion of neurogenesis	[111,112,113,115,116,117]
		protection against impairment of neurogenesis	[73,113,117]

## Abbreviations

2-APB	2-aminoethoxydiphenyl borate
4-AP	4-aminopyridine
5-HT1A receptor	serotonin 1A receptor
A2A receptor	adenosine A2A receptor
AIF	apoptosis-induced factor
Akt	protein kinase B
AMPA	α-amino-3-hydroxy-5-methyl-4-isoxazolepropionic acid
ATP	adenosine triphosphate
Bax	Bcl-2-associated X protein
Bcl-2	B-cell lymphoma 2 protein
bFGF	basic fibroblast growth factor
BrdU	5-bromo-2′-deoxyuridine
CaCl_2_	calcium chloride
CB1	cannabinoid receptor type 1
CB2	cannabinoid receptor type 2
CCL	chemokine (C-C motif) ligand
CHOP	C/EBP homologous protein
CNS	central nervous system
CoCl_2_	cobalt(II) chloride
CPZ	capsazepine
CRISPR	clustered regularly interspaced short palindromic repeats
CSPG	chondroitin sulfate proteoglycan
CUS	chronic unpredictable stress
DCX	double cortin
DMSO	dimethyl sulfoxide
EC_50_	half maximal effective concentration
EGTA	ethylene glycol-bis(2-aminoethylether)-*N,N,N’,N’*-tetraacetic acid
eiF2α	eukaryotic translation initiation factor 2α
ER	endoplasmic reticulum
EtOH	ethanol
FAAH	fatty acid amide hydrolase
FDA	U.S. Food and Drug Administration
GABA	γ-aminobutyric acid
GAP43	Growth-associated protein 43
GCV	ganciclovir
GFAP	glial fibrillary acidic protein
GFP	green fluorescent protein
GPCR	G protein-coupled receptor
GPR18	G protein-coupled receptor 18
GPR55	G protein-coupled receptor 55
HEPES	hydroxyethyl piperazine ethane sulfonic acid
hGMSC	human gingival mesenchymal stem cell
HO	heme oxygenase
hPLMSC	human periodontal ligament-derived mesenchymal stem cell
Iba-1	ionized calcium-binding adapter molecule 1
IC_50_	half maximal inhibitory concentration
IFNγ	interferon γ
ihPSC	induced human pluripotent stem cell
IL	interleukin
iNOS	inducible nitric oxide synthase
iPSC	induced pluripotent stem cell
IP_3_	inositol trisphosphate
KCl	potassium chloride
LDL	low-density lipoprotein
LPS	lipopolysaccharide
MAGL	monoacylglycerol lipase
MAP2	microtubule-associated protein 2
MARK1	mitogen-activated protein kinase 1
MEA	multi-electrode array
MgCl_2_	magnesium dichloride
mNCX	mitochondrial Na^+^/Ca^2+^ exchanger
mPTP	mitochondrial permeability transition pore
mTOR	mammalian target of rapamycin
MSC	mesenchymal stem cells
NaCl	sodium chloride
NADPH	nicotinamide adenine dinucleotide phosphate
NAGly	*N*-Arachidonylglycine
NF-κB	nuclear factor-κB
NMDAR	*N*-methyl-D-aspartic acid receptor
PAPR-1	poly(ADP-ribose) polymerase 1
PAX6	paired box protein
PI3K	phosphatidylinositol3-kinase
PKR	protein kinase R
PLC	phospholipase C
PPARγ	peroxisome proliferator-activated receptor γ
PrPres	protease-resistant prion protein
PSC	pluripotent stem cells
PTZ	pentylenetetrazole
S100B	S100 calcium binding protein B
SCN1A	sodium voltage-gated channel alpha subunit 1
SOCC	store-operated calcium channel
SOCE	store-operated calcium entry
TGF-β1	transforming growth factor β1
TH	tyrosine hydroxylase
TMEV	Theiler’s murine encephalomyelitis virus
TNF-α	tumor necrosis factor-α
TRPC	transient receptor potential cation channel subfamily C
TRPV	transient receptor potential cation channel subfamily V
VCAM-1	vascular cell adhesion protein 1
VGCC	voltage-gated calcium channel
Xbp	X-box protein
XT-1	xylosyltransferase 1
